# Combination of Insecticide Treated Nets and Indoor Residual Spraying in Northern Tanzania Provides Additional Reduction in Vector Population Density and Malaria Transmission Rates Compared to Insecticide Treated Nets Alone: A Randomised Control Trial

**DOI:** 10.1371/journal.pone.0142671

**Published:** 2015-11-16

**Authors:** Natacha Protopopoff, Alexandra Wright, Philippa A West, Robinson Tigererwa, Franklin W Mosha, William Kisinza, Immo Kleinschmidt, Mark Rowland

**Affiliations:** 1 Department of Disease Control, London School of Hygiene and Tropical Medicine, London, United Kingdom; 2 Department of Infectious Disease Epidemiology, London School of Hygiene and Tropical Medicine, London, United Kingdom; 3 Department of Health, District Medical Office, Muleba, Tanzania; 4 Kilimanjaro Christian Medical University College, Moshi, Tanzania; 5 National Institute for Medical Research, Amani Medical Research Centre, Muheza, Tanzania; 6 MRC Tropical Epidemiology Group, London School of Hygiene and Tropical Medicine, London, United Kingdom; University of Crete, GREECE

## Abstract

Indoor residual spraying (IRS) combined with insecticide treated nets (ITN) has been implemented together in several sub-Saharan countries with inconclusive evidence that the combined intervention provides added benefit. The impact on malaria transmission was evaluated in a cluster randomised trial comparing two rounds of IRS with bendiocarb plus universal coverage ITNs, with ITNs alone in northern Tanzania. From April 2011 to December 2012, eight houses in 20 clusters per study arm were sampled monthly for one night with CDC light trap collections. *Anopheles gambiae* s.l. were identified to species using real time PCR Taq Man and tested for the presence of *Plasmodium falciparum* circumsporozoite protein. ITN and IRS coverage was estimated from household surveys. IRS coverage was more than 85% in two rounds of spraying in January and April 2012. Household coverage with at least one ITN per house was 94.7% after the universal coverage net campaign in the baseline year and the proportion of household with all sleeping places covered by LLIN was 50.1% decreasing to 39.1% by the end of the intervention year. *An*.*gambiae* s.s. comprised 80% and *An*.*arabiensis* 18.3% of the anopheline collection in the baseline year. Mean *An*.*gambiae* s.l. density in the ITN+IRS arm was reduced by 84% (95%CI: 56%-94%, p = 0.001) relative to the ITN arm. In the stratum of clusters categorised as high anopheline density at baseline EIR was lower in the ITN+IRS arm compared to the ITN arm (0.5 versus 5.4 per house per month, Incidence Rate Ratio: 0.10, 95%CI: 0.01–0.66, p-value for interaction <0.001). This trial provides conclusive evidence that combining carbamate IRS and ITNs produces major reduction in *Anopheles* density and entomological inoculation rate compared to ITN alone in an area of moderate coverage of LLIN and high pyrethroid resistance in *An*.*gambiae* s.s.

## Introduction

In the past decade insecticide treated net (ITN) distribution and indoor residual spraying (IRS) of houses have been scaled-up and implemented together in 31 countries across Africa [1]. The rationale to combine these two interventions is to increase coverage and accelerate reduction of malaria compared to one of these interventions implemented alone. The combination can compensate for the loss of effectiveness when one intervention is compromised for example when there is insecticide resistance; when IRS is waning of [2], or due to ITN low usage or tears and wears [3]. The combined use of two insecticides has also been suggested as a potential tool for resistance management [4]. There is however inconclusive evidence that the combination will have an additive effect [5].

Small-scale experimental hut studies have demonstrated that the combined use of pyrethroid insecticide in both IRS and Long Lasting Insecticidal Nets (LLINs) fails to provide improvements in vector control or individual protection [6;7]. Improved control requires the combined use of insecticides from different classes such as pyrethroids for LLIN and carbamates or organophosphates for IRS [7–9]. Only community-level cluster randomised trials can demonstrate the potential for transmission control. Two cluster randomized trials have been conducted recently, the first using the carbamate bendiocarb for IRS in Benin [10] and the second using DDT in The Gambia [11]. Neither showed any additional benefit from combining IRS with ITN compared to ITN alone. Some non-randomised observational studies have produced evidence of an additional benefit, as in Equatorial Guinea [12] and Kenya [13], while others have not showed such an effect, as in Burundi [14]. Factors such as variation in malaria transmission intensity, vector behaviour, intervention coverage and insecticide resistance have been proposed to explain the discordant outcomes between trials [5]

In Tanzania, the national policy has been to scale up LLIN coverage, by first targeting children under five in 2009 followed by universal coverage of the entire population at risk in 2011. In some high transmission districts around Lake Victoria IRS with pyrethroid has been done annually since 2006. The present trial has been conducted in Muleba district where IRS and LLIN have been implemented together. The protective effectiveness of combining IRS and LLIN compared to LLIN alone on malaria infection prevalence in this trial was reported previously [15]. In this paper we report the effect of the intervention on vector density and entomological inoculation rate (EIR) as a proxy for malaria transmission.

## Material and Methods

### Study area

The study was conducted in Muleba district (1°45’S, 31°40’E), in North-West Tanzania on the western shore of Lake Victoria. The study area has been described in detail elsewhere [16]. Muleba covers an area of 3,550 km^2^ and has a population of 540,310 people (Tanzanian National Bureau of Statistics 2012 Census). The annual rainfall ranges from 850 mm to 1500 mm (Igabiro weather station 2006 to 2012) and is divided in a long rainy season from March to May and a short rainy season in October-December. Altitude in the study area ranges from 1100 to 1600 meters. The organisation RTI International conducted annual IRS in Muleba district from 2006 to 2011 with the pyrethroid lambdacyhalothrin (ICON 10CS, Syngenta, Basel, Switzerland). As part of the National Malaria Control strategy, net coverage has increased since 2005 through LLINs distribution campaigns initially targeting pregnant women and children under five in 2009 (63% of households provided with nets) and then targeting the entire population in 2011 (91% household ownership) [16]. There are two malaria transmission seasons following the long and the short rains. The prevalence of malaria during the baseline year of this trial was 9.3% in February 2011 and 22.8% in June 2011 [17]. High pyrethroid resistance in *An*.*ga*mbiae s.s. has been observed in this area [18].

### Study design and Intervention

This study presents the entomological outcomes of a cluster randomized controlled trial, conducted in 2011–2012, which compared the combined intervention of ITN and IRS versus ITN alone on malaria infection prevalence in children [15]. Every household in 109 villages was mapped to form 50 clusters with buffer zones at least 1 kilometer wide surrounding core sampling areas of two kilometers diameter in which the surveys were conducted. The core and buffer zones of each cluster received the same interventions. Half of the clusters were randomly allocated to receive IRS in addition to the pre-existing universal coverage of ITN. RTI International was responsible for conducting the two rounds of IRS using bendiocarb (FICAM 80% wettable powder, Bayer Leverkusen, Germany) in the 25 intervention clusters in January and April 2012. The decision to use bendiocarb instead of the pyrethroid lambdacyhalothrin (which was used in previous years) was taken by the national malaria control programme and PMI after high resistance to pyrethroids was reported in the area [18].

Five cross sectional malaria indicator surveys were conducted, two during the 2011 baseline year and three in the post intervention year. Results are reported elsewhere [15].

### Entomological monitoring

From April 2011, a series of 19 monthly entomological cross sectional surveys (seven in the baseline and twelve in the intervention year) were undertaken in the 20 clusters that were randomly selected in each arm of the study for the measurement of entomological outcomes. Each month, in eight randomly selected houses from each cluster, mosquito density was monitored using CDC light traps during one night. After obtaining informed consent from the family, a light trap was installed at the foot of a bed occupied by a family member sleeping under a treated or untreated bed net. Information was collected on house structure (type of wall and roof, presence of open eaves, ceiling and window screens), presence of livestock inside or outside the house, whether the house was sprayed, and bed net ownership and usage. Houses were randomly sub-sampled from the list of houses that had been sampled for the pre-ceding cross-sectional household survey. Mosquito collections were identified to species using a simplified morphological key adapted from Gillies and Coetzee [19], and subsequently tested by ELISA for detection of *Plasmodium falciparum* circumsporozoite protein (Pf-CSP) [20]. A sub-sample of *An*.*gambiae* s.l. was tested using Real time PCR Taq Man assay to distinguish between the two sibling species *An*.*gambiae* s.s. and *An*.*arabiensis* [21].

### IRS quality monitoring

The quality and coverage of IRS was investigated using the carbamate Insecticide Quantification Kit in the intervention year (IQK^TM^ [Innovative Vector Control Consortium, www.ivcc.com]). Wall scrapings of prescribed area were taken from the living rooms and bedrooms of 368 households in April 2012 and 490 household in June 2012, respectively four months after the first spraying and one month after the second spraying. The concentration of bendiocarb residue on the wall was assessed by a colorimetric assay which reacts to the presence of insecticide [22].

### Statistical analysis

Data were collected using PendragonTM Forms (Pendragon Corporation Software, Libertyville, USA) on Personal Digital Assistants (PDA) and analysed using STATA^TM^ (STATAcorp, Texas, USA, version 11.2). The EIR per month was calculated as the product of sporozoite rate and *An*.*gambiae* s.l. collected in the light trap for each house for each month. Baseline vector density was categorised, into low density clusters, mean *An*.*gambiae* s.l. < = 1 per household per night, and high density clusters mean *An*.*gambiae* s.l. >1 per household per night, to investigate interactions. *Anopheles* and Culex density ratios and EIR incidence rate ratios between the intervention and control arms were estimated using negative binomial regression adjusting for baseline low and high *Anopheles* density. Logistic regression was used to estimate sporozoite odds ratios. Standard errors were adjusted to allow for within cluster correlation of responses using robust standard errors (svy command in STATA, [23, 24]).

### Ethics statement

The trial was approved by the ethics review committees of the Kilimanjaro Christian Medical College, the National Institute for Medical Research Tanzania and the London School of Hygiene and Tropical Medicine (application no. 5814). The trial is registered with ClinicalTrials.gov (registration number NCT01697852).

## Results

### Baseline

In the baseline year 2,312 houses were surveyed between April and December 2011 of which on average 94.4% (N = 2183) consented to participate (range 92.4–96.8% between rounds). Of the remainder 0.1% refused to participate, 4.2% of houses were vacant, 0.2% were not located, 0.4% not visited and 0.6% were ineligible (no children under 15 years).

15,860 mosquitoes were collected during the seven baseline rounds of which 45.3% were Anopheline and 54.5% Culicine. 98.3% of Anophelines were *An*.*gambiae* s.l. and 0.2% *An*.*funestus*. *An*.*gambiae* s.s. represented 81.4% (95%CI: 75.5–86.1) and *An*.*arabiensis* 18.6% (95%CI: 13.9–24.6) of the total collection of *An*.*gambiae* s.l. The sporozoite rate in *An*.*gambiae* s.l. during the baseline year was 1.4% (95%CI: 1.1–2.0) and only *An*.*gambiae* s.s. were found positive.

In the baseline year, household characteristics, coverage of vector control interventions (Table 1) and entomological outcomes (Table 2) were similar between the control and intervention arms. The majority of houses had metal roofs, mud walls, open eaves and were unscreened. The average household had 6 individuals; 2–3 sleeping places and kept livestock.

**Table 1:** Baseline characteristics of household in the study area.

Characteristics	All			ITN arm			ITN + IRS arm		
	N	% /Mean	[95%CI]	N	% / Mean	[95%CI]	N	% / Mean	[95%CI]
% of iron/tin roof house	2182	77.4%	[70.8,82.8]	1062	80.3%	[71.3–87.0]	1120	74.6%	[64.8–82.5]
% of mud house	2175	65.5%	[58.9–71.6]	1061	61.8%	[53.1–69.8]	1114	69.0%	[59.2–77.4]
% of houses with open eaves	2159	64.2%	[59.7–68.6]	1051	61.8%	[55.5–67.8]	1108	66.5%	[60.1–72.4]
% households owning livestock	2183	77.9%	[74.9–80.7]	1062	75.1%	[70.0–79.7]	1121	80.6%	[77.6–83.2]
% of screened house	2178	1.6%	[1.0–2.5]	1060	2.1%	[1.4–3.2]	1118	1.2%	[0.4–3.0]
Type of houses	2183			1062			1121		
*Better constructed*		16%	[12.6–20.2]		16.9%	[12.2–23.1]		15.2%	[10.6–21.2]
*Medium*		61.3%	[55.4–66.0]		63.4%	[56.9–69.4]		59.4%	[52.1–66.3]
*Poorly constructed*		22.6%	[17.2–29.2]		19.7%	[13.0–28.7]		25.4%	[17.6–35.2]
Mean number of people per household	2182	5.6	[5.5–5.7]	1061	5.5	[5.3–5.7]	1121	5.7	[5.5–5.8]
Mean number of sleeping space	2181	2.4	[2.3–2.5]	1061	2.4	[2.2–2.5]	1120	2.4	[2.3–2.6]
Average altitude in meters	2182	1341	[1301–1381]	1062	1332	[1273–1391]	1120	1349	[1290–1407]
Average number of time sprayed (range)	2175	3.2	(0–5)	1056	3.0	(0–5)	1119	3.4	(0–5)
% houses sprayed in 2011	2182	94.6%	[92.7–96.1]	1062	92.7%	[89.6–95.0]	1120	96.4%	[94.2–97.8]
% of household that own at least 1 LLIN	2180	94.7%	[93.3–96.1]	1060	93.0%	[90.7–95.4]	1119	96.3%	[94.7–97.9]
% of household with all sleeping space coverd by LLIN	2176	50.1%	[45.1–55.1]	1059	47.8%	[40.5–55.1]	1117	52.4%	[45.0–59.8]

**Table 2:** Entomological indicators mean culex and *An*.*gambiae* s.l. per house, sporozoite rate and entomological inoculation rate, in baseline year.

	ITN arm			ITN + IRS arm		
Outcome	Total Houses	Mean	[95%CI]	Total Houses	Mean	[95%CI]
Mean Culex/house/night	1055	3.8	[2.3–6.1]	1120	2.7	[1.8–4.1]
Mean *An*. *gambiae* s.l/house/night	1055	3.1	[1.0–9.6]	1120	2.2	[0.5–9.1]
EIR/house/month^1^		1	[0.4–2.8]		1.3	[0.4–4.4]
	Total *Anopheles* tested	%	[95%CI]	Total *Anopheles* tested	%	[95%CI]
Proportion *An*. *Arabiensis* ^2^	1018	22.1%	[16.9–28.3]	1452	14.1%	[10.5–18.5]
Sporozoite rate^2^	1359	1.1%	[0.8–1.4]	1466	2.0%	[1.4–2.7]

^**1**^Entomological Inoculation Rate per month per house per month = Infectious bites per month calculated as SR*Anopheles density*30 day

^**2**^ weighted by the inverse of the sampling fraction

**Fig 1:**
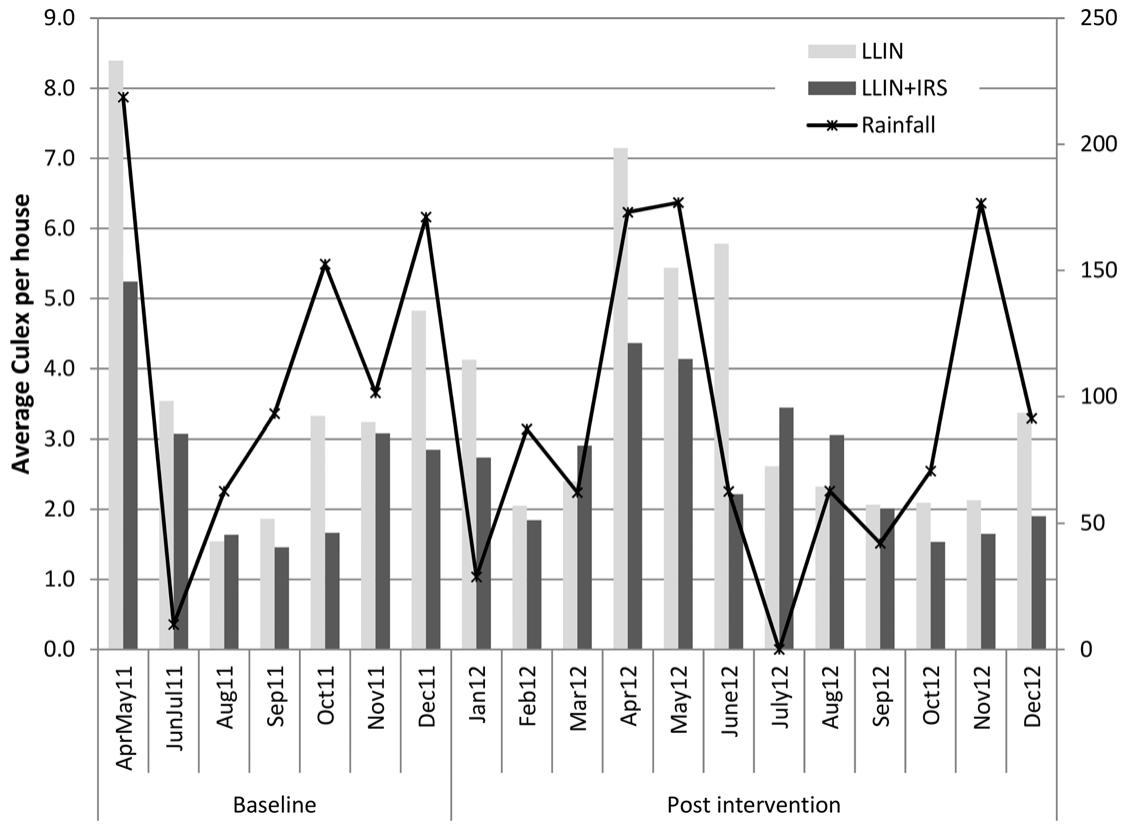
Monthly mean *Anopheles* density per house in the two arms and rainfall during baseline and post intervention period.

The average number of mosquitoes per household per night was 6.9 (95%CI: 3.4–13.9) in LLIN arm and 5.0 (95%CI: 2.2–11.6) in the IRS+ITN arm (Table 2). Sporozoite rate was higher in the intervention arm; however there was no difference in monthly Entomological Inoculation Rate (EIR) between the two arms at baseline. The average number of Anophelines per household per night varied during the year (Fig 1), with the highest density (mean = 13.1) observed during the first rainy season (April-May) and the lowest (mean = 0.13) during the dry season in August. Apart from temporal variations, there was considerable heterogeneity between clusters with 28 clusters having one or less Anopheline collected per house per night, nine clusters between one to three *Anopheles* and three clusters with more than 20.

### Post intervention

Twelve rounds of mosquito surveillance were completed between January and December 2012. 91.6% (3,834) households of the 4,187 surveyed gave consent (range: 87.1–96.1% between rounds). 0.4% of households refused and 6.5% were vacant.

Householder surveys recorded an IRS coverage of 89.3% during the January campaign, ranging from 65.6% to 100% between clusters. IRS coverage after the April campaign was 86.9%, ranging from 51.6% to 100% between clusters. Four months after the first spray round, 56.4% (95%CI: 48.0–64.8) of the rooms had the recommended level of insecticide on their walls according to IQK tests. One month after the second round the room coverage was, 84.6% (95%CI: 79.7–89.5).

LLIN ownership (defined as at least one LLIN per house) after the LLIN universal coverage campaign (UCC) in 2011 was 94.7% (baseline year). The proportion of households with all sleeping places covered by a LLIN, was 50.1% (95%CI: 54.4–63.4) after the UCC but decreased to 39.1% (95%CI: 34.0–44.2) by the end of the intervention period one and half year later.

A total of 15,480 mosquitoes were collected during the 12 rounds of surveillance post intervention. 26.1% were Anopheline and 97% of these were identified as *An*.*gambiae* s.l.. Important variation in *Anopheles* density was observed between seasons in the control arm with the highest density following the long and short rainy season in April-May and November December; seasonal variation was also seen in the intervention arm but with lower amplitude (Fig 1). 20.7% (95%CI 13.4%-28.1%) of the *An*.*gambiae* s.l. collected were *An*.*arabiensis* the remaining being *An*.*gambiae* s.s.

**Fig 2:**
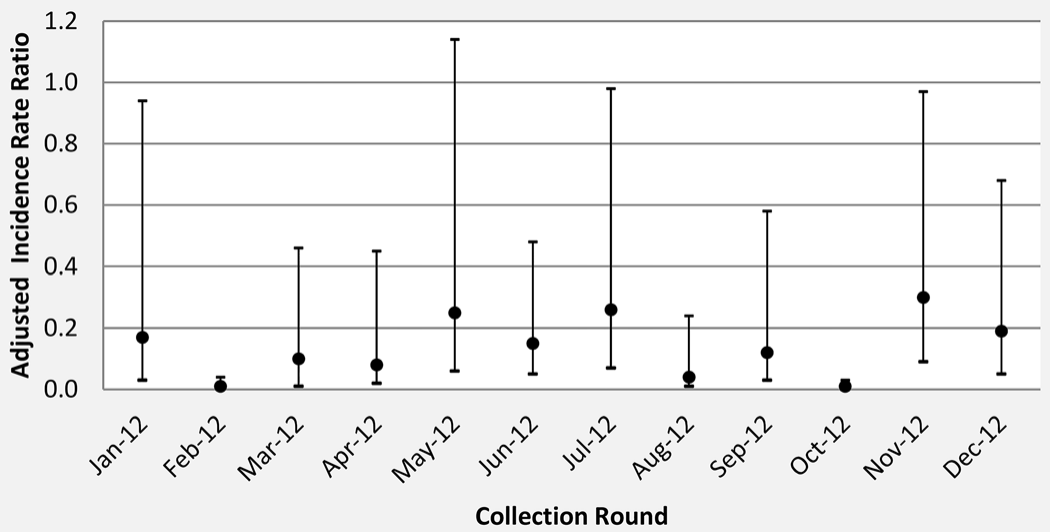
Adjusted incidence rate ratio for mean *An*.*gambiae* s.l. density per household between ITN arm (reference IRR = 1) and ITN+ IRS arm per light trap collection round.

All the entomological outcomes were lower in the intervention arm compared to the control arm (Table 3). There was strong evidence that *An*.*gambiae* s.l. density was 84% (95%CI: 56%-94%, p = 0.001) lower in the ITN+IRS arm relative to the ITN arm when adjusted for the baseline density of *An*.*gambiae* s.l.. There was no evidence that the effect of the intervention on *An*.*gambiae* s.l. density differed between the high and low *Anopheles* density clusters (interaction p-value = 0.506). The effect was consistent in every collection month, with the greatest differences observed in February to April and August to October (Fig 2). There is strong evidence that monthly EIR was lower in the intervention arm (EIR = 0.2; IRR = 0.01; pvalue<0.001) compared to the control arm (EIR = 1.2) with evidence that the intervention effect was higher in the low compared to the high vector density clusters (interaction pvalue<0.001). There was no evidence that the intervention reduced the overall sprorozoite rate (OR = 0.73, p = 0.607). However, no sporozoites were found in *Anopheles* collected in the IRS+ITN arm in the low density stratum of the study, compared with 4.2% sporozoite rate in the LLIN arm of this stratum and sporozoite rate was similar in the high density stratum (interaction pvalue<0.001).

**Table 3:** Post intervention entomological outcomes by study arm and by low and high density *An*.*gambiae* s.l. density stratum at baseline.

		ITN arm		ITN + IRS arm					Interaction
Outcome		N	%/Mean	N	%/Mean	Ratio	95%CI	P	P value^4^
Sporozoite rate (SR)									
	All clusters	3059^2^	2.5%	717	1.8%	OR^1^ = 0.73	0.21–2.54	0.607	
	Clusters with 1 or less *An*. *gambiae* s.l/h/night at baseline	248	4.4%	41	0.0%	-	-	-	<0.001
	Clusters with more than 1 *An*. *gambiae* s.l/h/night at baseline	2811	2.3%	676	1.9%	OR = 0.82	0.19–3.54	0.766	
*An*. *gambiae* s.l density /household/night									
	All clusters	1905^3^	1.7	1910	0.4	DR^1^ = 0.16	0.06–0.44	0.001	
	Clusters with 1 or less *An*. *gambiae* s.l/h/night at baseline	1522	0.2	1145	0.03	DR = 0.22	0.09–0.50	0.001	0.506
	Clusters with more than 1 *An*. *gambiae* s.l/h/night at baseline	383	7.8	765	0.9	DR = 0.12	0.02–0.75	0.028	
EIR/household/month									
	All clusters adjusted	1901^3^	1.2	1906	0.2	IRR^1^ = 0.01	0.00–0.08	<0.001	
	Clusters with 1 or less *An*. *gambiae* s.l/h/night at baseline	1519	0.2	1143	0	-	-	-	<0.001
	Clusters with more than 1 *An*. *gambiae* s.l/h/night at baseline	382	5.4	763	0.5	IRR = 0.10	0.01–0.66	0.021	

^1^Odd Ratio (OR), Density Ration (DR) and Incidence Rate Ration (IRR) are adjusted for baseline mean cluster An. gambiae s.l. as categorical with < = 1 An. gambiae s.l./household/night and clusters>1

^2^N = Total Anopheles for Sporozoite rates and

^3^N total of houses for An. gambiae s.l. density and EIR

^4^ Interaction p value = p-value for difference between strata

Comparing the two sibling species, *An*.*gambiae* s.s density was 85% lower (adjusted IRR: 0.15, 95%CI: 0.05–0.44, p = 0.001) in IRS+ITN arm compared to the ITN arm, while there was no evidence for a reduction in *An*.*arabiensis* (IRR: 0.46, 95%CI: 0.11–1.89, p = 0.276). Sporozoite rate in the *An*.*arabiensis* was 0.7% (95%CI: 0.1%-4.1%, N = 137 tested) in the ITN arm and nil in the IRS+ITN arm (N = 102).

There was no evidence for a difference in Culex species density between the two arms. The average number of Culex per house per night was 3.4 in ITN arm and 2.7 in the combined ITN+IRS arm (IRR: 0.78, 95%CI: 0.37–1.65, p = 0.51).

## Discussion

This community randomised control trial provided evidence that in an area of northern Tanzania two rounds of carbamate IRS combined with a moderate coverage of ITN produced a reduction in Anopheline density and entomological inoculation rate of public health importance compared to use of ITN alone; this effect explains the decrease in malaria infection prevalence reported previously from this trial [15]. The six-fold reduction in EIR during the course of the intervention translated into a two-fold reduction in infection prevalence [15]. The reduction in *Anopheles* density was still evident during the second transmission period eight months after the second spraying and long after the residual activity of the insecticide had waned [22]. This may be explained by the reduction of vector population after IRS to a point that it had still not recovered. In addition ITNs might have delayed vector population recovery as observed in a study in Equatorial Guinea [2].

The combination had no additional impact on *An*.*arabiensis* density. During the present trial, the relative proportion of *An*.*arabiensis* to *An*.*gambiae* s.s. was 18% in the indoor light trap collections while *An*.*arabiensis* formed only 1% of the collection resting on the walls [18]. This indicates that the local *An*.*arabiensis* was less endophilic than *An*.*gambiae* s.s and might have reduced contact with insecticide on walls. Several studies and mathematical models [25] have reported or predicted a reduced impact of IRS or ITN on *An*.*arabiensis* due to its outdoor feeding and resting behaviour and this has been proposed to explain the shift in species ratio towards *An*.*arabiensis* observed in some areas [26]. The perception is that IRS or ITN will reduce *An*.*gambiae* s.s. to low densities but residual transmission by *An*.*arabiensis* will still continue, *An*.*arabiensis* being less tractable to IRS or ITN interventions. During the present study, there were no collections to assess any outdoor or residual transmission potential. However on the basis of the EIR estimates, *An*.*arabiensis* would seem to contribute little to indoor malaria transmission in this region of Tanzania.

WHO susceptibility tests with bendiocarb revealed that during the intervention there was an increase of phenotypic carbamate resistance from a baseline of 16% to 69% eight months after the second round of IRS [27]. Bendiocard IRS seems to have exerted a strong selection pressure on local vectors. In Benin, however increase in bendiocarb resistance was attributed the use of insecticide for other purposes [28].The mechanism of resistance and its strength in Muleba have not yet been determined.

Several small scales, experimental hut studies have examined the potential impact of combining IRS and LLIN on vector mortality and individual protection from mosquito bites. In the West African country of Benin, the addition of pyrethroid IRS to pyrethoid LLIN did not increase *Anopheles* mortality or personal protection relative to LLIN alone [6]. Similarly, no increase in mortality was observed by the addition of lambdacyhalothin or DDT IRS to LLIN in Tanzania [7]. Only when the partner IRS was from another class of insecticide to the pyrethroids, such as the organophosphate pirimiphos methyl [7] or the carbamate bendiocarb [8] or the pyrrole chlorfenapyr [9] was a significant increase in mortality observed compared to LLIN alone [8]. However the full benefit of combining IRS and ITN can only be evaluated through community randomised trials.

Apart from the present study, two recently published randomised control trials have evaluated the additional impact of combining IRS and ITN on entomological outcomes. In the Gambia there was no evidence that the addition of IRS with DDT to high coverage LLIN reduced *Anopheles* density or the EIR [11]. The difference between the present trial and the Gambian trial could either be due to the higher coverage of LLIN achieved in The Gambia (over 90%), the absence of pyrethroid resistance observed, or potential spill-over effects between the two study arms. In another randomized controlled trial in Benin no additional effect on human biting rate or EIR was observed when IRS with bendiocarb was combined with LLIN [10]. As in Tanzania, Benin has reported high pyrethroid resistance and moderate coverage (65%-70%) of LLIN achieved but unlike in Tanzania only one round of IRS was done and considering the short residual activity of bendiocarb, efficacy would be limited and no effect was observed compared to the LLIN arm. A fourth study in Sudan showed no significant additional protection when the two interventions were combined [5]. LLIN coverage was high in Sudan, but *An*.*arabiensis* was the main vector and as in Tanzania IRS might be less effective against this vector.

In the study area, pyrethroid resistance ranged from 60% to 92% during the trial [18;27] and this might have hampered the effectiveness of the LLIN. Because LLIN usage was only moderate, IRS was probably compensating for the limited community impact achieved by the ITN. However, a per protocol analysis indicated that the individuals who used LLINs had lower infection risk compared to those not using a LLINs and this regardless if their house was sprayed or not [29]. This suggests that LLIN was still effective and did provide an additional protection despite high coverage of IRS.

In conclusion the present trial provides evidence that the combination of carbamate IRS and pyrethroid ITN produces a marked reduction in *Anopheles* density and entomological inoculation rate compared to ITN alone and this effect can account for the 57% reduction in malaria infection prevalence observed in the combination arm of this trial.
