# Correction: Combination of Insecticide Treated Nets and Indoor Residual Spraying in Northern Tanzania Provides Additional Reduction in Vector Population Density and Malaria Transmission Rates Compared to Insecticide Treated Nets Alone: A Randomised Control Trial

**DOI:** 10.1371/journal.pone.0146629

**Published:** 2016-01-05

**Authors:** Natacha Protopopoff, Alexandra Wright, Philippa A West, Robinson Tigererwa, Franklin W Mosha, William Kisinza, Immo Kleinschmidt, Mark Rowland

The image for Fig 1 is incorrect. Please view the correct Fig 1 here.

**Fig 1:**
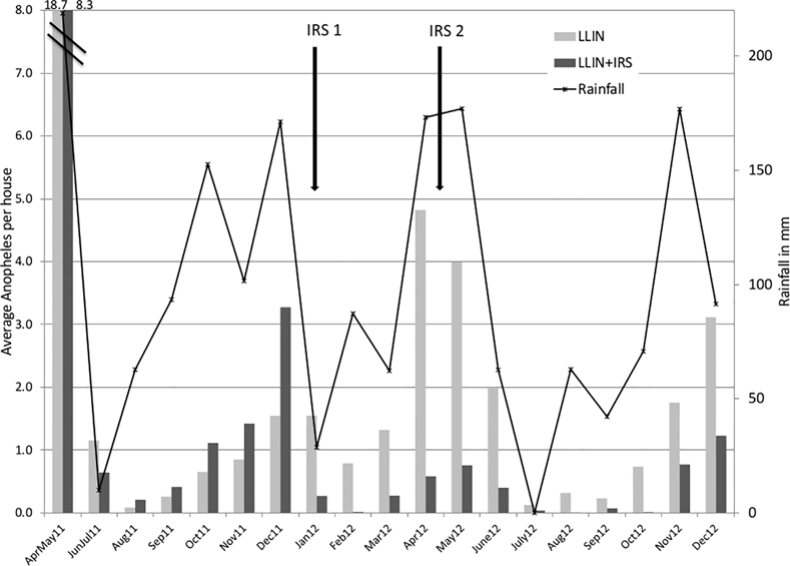
Monthly mean *Anopheles* density per house in the two arms and rainfall during baseline and post intervention period.
